# Optimal Use of Ganciclovir and Valganciclovir in Transplanted Patients: How Does It Relate to the Outcome?

**DOI:** 10.1155/2018/8414385

**Published:** 2018-09-17

**Authors:** Maryam Mozaffar, Shahrzad Shahidi, Marjan Mansourian, Shirinsadat Badri

**Affiliations:** ^1^Isfahan Pharmacy Students' Research Committee, Isfahan University of Medical Sciences, Isfahan, Iran; ^2^Isfahan Kidney Diseases Research Center, Isfahan University of Medical Sciences, Isfahan, Iran; ^3^Department of Biostatistics and Epidemiology, Isfahan University of Medical Sciences, Isfahan, Iran; ^4^Department of Clinical Pharmacy and Pharmacy Practice, Isfahan University of Medical Sciences, Isfahan, Iran

## Abstract

**Objective:**

Organ transplant recipients receive immunosuppressive regimens to prevent transplant rejection, which put them at increased risk for opportunistic infections like cytomegalovirus (CMV). Ganciclovir and Valganciclovir are mostly used to prevent or treat CMV. Any incorrect use of the drug may have serious consequences for patients. In this study, the outcome of transplant recipients was assessed in relation to the optimal or suboptimal use of Ganciclovir or Valganciclovir.

**Methods:**

This study was performed on 148 hospitalized patients who received Ganciclovir or Valganciclovir in the nephrology and kidney transplantation departments of our university hospitals, from March 2012 to December 2016. Patients' demographic and clinical data including dose and duration of treatment were collected and then analyzed in comparison with the standard CMV treatment protocols.

**Findings:**

About 94.6% of patients received Ganciclovir or Valganciclovir therapy consistent with the standard defined indications. The mean ratio of prescribed daily dose to the optimal dose was 2.9 in the first dose, 2.0 in the second dose, 1.3 in the third dose, and 1.5 in the fourth dose. From 148 included patients, 26.5% experienced CMV infection once, 7.2% experienced CMV infection twice, and 1.2% had CMV infection for 3 times, within six-month follow-up after first episode of antiviral therapy during hospitalization.

**Conclusion:**

In this study, empiric anti-CMV therapy was initially given. The doses used were generally higher than recommended but we could not find more adverse events in the patients receiving high initial doses. In any case, it seems necessary to advocate use of standard treatment guidelines to avoid adverse outcomes.

## 1. Introduction

Medication use evaluation (MUE) is an ongoing, systematic process generally managed criteria, based by an interdisciplinary team [1]. MUE involves a comprehensive review of practitioner prescribing, pharmacist dispensing, and patient use of medication [2]. The main objective is to promote effectiveness and safety of drug therapy; as a result, optimal medication use can improve patient outcomes and minimize overall costs [3–5]. MUE findings may help managed healthcare systems to improve prescribing patterns and formulary compliance, while optimizing use of scarce resources [6].

For patients with end-stage renal disease (ESRD), kidney transplantation is the best substitution option. However, the immunosuppressive regimen put the patients at high risk for infection with opportunistic pathogens, such as cytomegalovirus (CMV) [7]. The virus spreads in population and becomes latent until immune system suppressed, while it reactivates [8, 9]. In transplantation, CMV can be transmitted through graft or blood transfusion [10].

CMV infections outbreak in three types: primary infection, reactivation, and super infection [7]. CMV infection is defined as the virus detection in blood without clinical symptoms, but CMV disease is viremia with clinical symptoms ranging from mononucleosis-like viral syndrome to less common tissue-invasive disease [11, 12]. Degree of immunosuppression determines if infection leads to clinical disease or not [13]. CMV disease presents with clinical manifestations, as well as indirect effects including bacterial or fungal superinfections, acute or chronic allograft rejection, and allograft loss [14]. Risk factors related to CMV disease are immunosuppressive regimen and donor (D)/recipient (R) CMV serostatus based on immunoglobulin G (IgG) antibody to CMV [15]. Preventive strategies include universal prophylaxis in which antiviral therapy is administered for six months to all patients at risk and preemptive therapy in which antiviral therapy initiates when laboratory tests show early CMV replication [15, 16]. Findings suggest that it is better to use universal prophylaxis for high-risk patients (D+/R-) and preemptive therapy for moderate risk (D+/R+, D-/R+) or low risk patients (D-/R-) [17, 18]. Nowadays, Ganciclovir and Valganciclovir are first-line antiviral drugs to prevent or treat CMV infection [19]. Foscarnet, cidofovir, and leflunomide are second-line drugs used for Ganciclovir-resistant CMV infections [14].

There are approved protocols to prevent or treat CMV disease but because of the obstacles including economic shortcomings, insurance unaffordability, and medication nonavailability, some transplant centers cannot completely follow these protocols in the mentioned circumstances. Empiric therapy that is used alternatively in some transplant centers may increase the risk of CMV resistance and relapse [20]. In addition, any incorrect use of the drug (including dosage and duration of treatment) will have serious consequences for patients. No comprehensive study has yet been done to assess the optimal use of these drugs in relation to the patient's outcome. It seems that the results of such study would be of concern in the prescription process improvement and rational use of these drugs in accordance with approved protocols.

## 2. Method

This study was designed as a prospective observational research to evaluate the usage pattern of Ganciclovir and Valganciclovir by conducting a MUE program in the nephrology and kidney transplantation departments of our university hospitals, from March 2012 to December 2016.

The study was performed on all hospitalized transplanted patients (aged ≥ 18 years old) for whom intravenous Ganciclovir or oral Valganciclovir was prescribed during the study period. A pharmacist performed data collection in the wards, by reviewing patients' medical documents and charts and laboratory findings. The purpose was to study the current pattern of intravenous Ganciclovir and oral Valganciclovir administration while monitoring the practitioner prescribing practice, from the perspective of observance of all the necessary rules in administering, dosing and monitoring the antiviral therapy. Then the researcher followed these patients for six months, using patient's files in physicians' offices to evaluate the impact of drug therapy on patient's outcomes. The purpose was to evaluate the effect of accuracy of therapy with Ganciclovir and Valganciclovir (correct indication, drug dosage, duration of treatment, etc.) on the patient's outcome mainly considering the failure of antiviral therapy, recurrence or relapse of CMV infection, organ rejection or survival, and antiviral medications' side effects.

The data collection form was designed as Ganciclovir use evaluation checklist which was prepared by the researcher after related literature review and expert consultation (nephrologist and clinical pharmacist). Assessment criteria were developed to evaluate the appropriateness of Ganciclovir and Valganciclovir use, by applying the recent standard treatment protocols for CMV infection in transplanted patients [19, 21].

MUE checklists were completed for all patients by the researcher (pharmacist) based on information derived from the patients' charts and visiting the patients by accompanying the physicians during daily medical rounds. Demographic and clinical data were retrieved from the patients' charts and laboratory tests. Of laboratory results, data on serum creatinine, blood urea nitrogen (BUN), hemoglobin, white blood cells (WBC), neutrophil count, red blood cells (RBC), and platelet count were recorded. The aim was to evaluate the trend of graft function, patients' clinical improvement, and monitoring the possible occurrence of any drugs' side effects by following the tests.

The on-admission variables included age, weight, and height, reason of organ failure, state of immunosuppression, medical history, and graft function. The pharmacotherapy indicators included transplantation drug regimen, indication for Ganciclovir and Valganciclovir use (CMV prophylaxis or treatment), and their dosage and frequency of administration. Outcome indicators included the occurrence or relapse of CMV infection, organ rejection or survival, and adverse effects of these medications; all were evaluated within a six-month follow-up after the patient's antiviral therapy during hospitalization. The collected data were categorized and descriptively analyzed using Statistical Package for Social Sciences software, version 20.0, for Windows (SPSS, Chicago, IL, USA). In every category of data, the number and percentage of cases in which drug therapy was in accordance with standard therapeutic protocols and predetermined criteria for optimal administration and usage of the mentioned drugs were calculated. Chi-square and Fisher's exact tests were applied to assess the relationship between the optimal or suboptimal practice of antiviral therapy regarding drugs' dosages and variables indicating the outcome. In all analysis,* P* value < 0.05 was considered significant.

## 3. Results

During the study period, 148 cases have been reviewed. The demographic information of patients are presented in Table 1. Three most frequent reasons of kidney failure were hypertension (18.9%), glomerulonephritis (16.2%), and diabetes mellitus (13.5%). From 148 included patients, 37 cases were receiving Ganciclovir or Valganciclovir for CMV prophylaxis besides their immunosuppressive regimen after transplantation. These consisted of patients who received antilymphocytic therapy as a part of their regimen soon after transplantation surgery during their hospitalization or for managing an episode of organ rejection. The other 111 cases were receiving these drugs for treating CMV disease diagnosed by the patient's clinical symptoms and its confirmation by CMV serology tests.

All patients were donor+/recipient+ for CMV IgG antibody indicating moderate risk for developing subsequent CMV infection and disease. In this population, we had no high-risk patients for CMV disease (D+/R-) considering CMV IgG antibody serostatus. In 11 patients (7.4%), the duration of treatment with Ganciclovir or Valganciclovir was within the range of 15 to 40 days. In 137 patients, the duration of antiviral therapy depended on the individual patient's risk factors and the state of immunosuppression, ranging from 3 days to 3 months. Some experts empirically recommend 21 days of prophylaxis with antiviral medication; in this study, 46.6% of patients received this regimen for a minimum of 21 days.

In 140 patients (94.6%), Ganciclovir or Valganciclovir was administered in accordance with the therapeutic indications (as prophylaxis therapy for CMV infection or as a treatment for CMV disease) mentioned in CMV treatment protocols [19, 21]. Considering creatinine clearance on the time of drug administration, the optimal dose of Ganciclovir or Valganciclovir were retrieved from the respective standard dosage guideline [19]. If the drug dose had been changed by the physician during treatment period, then the new optimal dose was obtained regarding the recent creatinine clearance at the time.  Table 2 shows administered and optimal doses of these two drugs in the studied population at different dosing times during patient's hospitalization. In 79 patients (53.4%), drug dosages had been changed at least once (maximum four times in some patients), while in 69 patients (46.6%), no dosage changes were done.

Appropriateness of the administered dose was determined at different dosing times for each drug. The administered dose is considered appropriate if it was within the range of 80-120% of the optimal dose. These data, as percent of the patients who received the appropriate dose, are shown in Table 2.

Considering the dosage of Ganciclovir and Valganciclovir used in this population, the mean ratio of the prescribed daily dose to the optimal daily dose was 2.9 in the first dose, 2.0 in the second dose, 1.3 in the third dose, and 1.5 in the fourth dose, considering prevention and treatment doses as a whole. This ratio was more than 1 in all dosing times, demonstrating that the administered dose was totally more than the optimal dose. Hence, being overweight was proposed as a possible reason, since the physician may consider the actual body weight (ABW), not the ideal (IBW) one, for medication dosing or calculating the creatinine clearance. So, the appropriateness of administered dose in comparison to the optimal dose was reassessed regarding overweight as a confounding factor. Overweight was defined as ABW > 120% of IBW. For all analysis at different dosing times,* P* value was more than 0.05, demonstrating that this factor may not have the main role in the overdosing practice.

As a consequence of inappropriateness of antiviral therapy, two components may be considered: occurrence or reoccurrence of CMV infection and antiviral medications' side effects. From 148 included patients, 26.5% experienced CMV infection once, 7.2% experienced CMV infection twice, and 1.2% had CMV infection for three times, within six-month follow-up after first episode of antiviral therapy during hospitalization. The mean interval between discontinuing of the antiviral drugs and the first CMV infection episode was 2.4 months.

In 9 patients, the graft was completely rejected which leads to nephrectomy and dialysis. In 3 patients, other organs infected with CMV (one case of cytomegalovirus pneumonia, one case of cytomegalovirus colitis, and one case of ophthalmic CMV). The case of cytomegalovirus pneumonia and two other CMV-infected patients died.

The occurrence or relapse of CMV infection was assessed in relation to the appropriateness of Ganciclovir or Valganciclovir dose. The occurrence or relapse of CMV infection was seen in 10.3% of patients who received the optimal dose of antiviral medications (within the range of 80-120% of the standard dose), while it was documented in 10.8% of patients who not received the optimal dose. The *P* value was more than 0.05 for all assessments at different dosing times.

The on-admission and on-discharge results of laboratory tests are demonstrated in Table 3. These data were followed to evaluate the trend of graft function, patients' clinical improvement, and monitoring the possible occurrence of any drugs' adverse effects. However, due to the poor documentation of adverse events during hospitalization and afterwhile in the physicians' offices, following the patients' outcome was not feasible with this regard. Regarding the antiviral medications' side effects, four patients experienced drug-induced leukopenia and four patients had thrombocytopenia, in whom the antiviral drugs were discontinued. There was not any relationship between the optimal or suboptimal dose of the medications and these mentioned adverse effects.

## 4. Discussion

This study was the first medication use evaluation on Ganciclovir and Valganciclovir in kidney transplanted patients. In this study, the mean ratio of prescribed daily dose to the standard dose shows that antiviral drugs are totally used more than the recommended daily dose which can emerge drug resistance and increase the cost of treatment and adverse drug effects [22]. This ratio was the highest in the first dose which indicates an incorrect pattern of prescription in many patients. In this pattern, the physician prescribed a high dose of Ganciclovir or Valganciclovir at the first dose, likely because of overestimation of the kidney function, and did not adjust it through the course of treatment. So, the patients bear unnecessary costs and adverse drug effects at first doses, while at the end of treatment course, drug dosage is less than sufficient amount and may cause infection [23].

Duration of CMV prophylaxis mostly depends on donor and recipient serostatus. Unavailability and the cost of medications are the most prevalent causes of short treatment period in our study. Routine prophylaxis is not recommended for low risk patients (D-/R-) [19]. In our study, the duration of antiviral therapy depends on physician's opinion and patient's clinical situation.

We followed up patients regarding CMV infection after transplantation, in order to investigate the graft and patient survival. Ganciclovir only prevents viral replication, so the occurrence or relapse of infection or CMV disease would be expected after successful treatment. Infection recurrence may not affect patient's survival significantly but it increases morbidity and costs [22]. The mean interval between discontinuing of the antiviral drugs and the first episode of CMV infection or reinfection was 2.4 months. This result was in consistent with previous studies in which the risk of CMV infection was reported to be higher in the first three months after transplantation [21].

Our study results did not show any significant relationship between the correctness of Ganciclovir or Valganciclovir dosing and the patients' outcome. This unexpected finding could be explained by this point that drugs dosages were not optimal in the majority of patients. This may encounter many of these patients with the same outcome regarding the consequence of antiviral therapy; so due to the uniformity of the wrong practice in the studied patients, any significant differences in the outcome went hidden within the population. This study suffers a significant limitation, i.e., poor documentation and availability of patients' clinical data in some physicians' offices, which makes it difficult to thoroughly record the CMV monitoring data and any drugs' adverse effects.

In this study, empiric anti-CMV therapy was initially given. The doses used were generally higher than recommended but we could not find more adverse events in the patients receiving high initial doses. In any case, it seems necessary to advocate use of standard treatment guidelines to avoid adverse outcomes. Clinical pharmacists can ease this process by addressing the areas to be improved, solving drug related problems and increasing medication adherence [23].

## Figures and Tables

**Table 1:** Patients' demographic and clinical characteristics (N = 148).

**Characteristics (unit) **	**Value **
Age (years)	37.8 ± 15.5
Sex (M/F)	90 (60.8%) / 58 (39.2%)
Actual body weight (kg)	62.3 ± 24.6
Height (cm)	163.4 ± 17.6
Ideal body weight (kg)	58.1 ± 11.9
Overweight (ABW > 120% of IBW)	36 (24.3%)
Donor (alive/cadaveric)	106 (71.6%) / 42 (28.4%)
First/Second transplantation	128 (86.5%) / 20 (13.5%)
CMV serostatus (CMV IgG antibody)	
Moderate risk (D+/R+)	148 (100%)

Data presented as mean ± SD or number (%) of the patients.

CMV: cytomegalovirus; IgG: immunoglobulin G; SD: standard deviation; M: male; F: female; ABW: actual body weight; IBW: ideal body weight; D/R: donor/recipient.

**Table 2:** Doses of Ganciclovir and Valganciclovir in the studied population at different dosing times during patient's hospitalization.

**Doses at different dosing times** *∗*	**Prophylaxis of CMV infection** ^**£**^ (N = 37)		**Treatment of CMV disease ** (N = 111)	
	**Ganciclovir**	**Valganciclovir**	**Ganciclovir**	**Valganciclovir**
First administered dose	2.1 ± 1.3	411.0 ± 117.8	2.8 ± 1.6	512.1 ± 184.5
First optimal dose	1.2 ± 0.9	285.0 ± 183.4	1.8 ± 1.2	400.2 ± 201.7
Appropriateness of administered dose compared to optimal dose *∗∗*	18.1%	38.5%	24.8%	43.2%
Second administered dose	2.4 ± 1.5	456.4 ± 206.2	3.1 ± 0.9	571.9 ± 214.8
Second optimal dose	2.0 ± 1.1	409.0 ± 193.4	2.7 ± 1.8	510.0 ± 321.3
Appropriateness of administered dose compared to optimal dose	29.5%	47.5%	32.4%	41.3%
Third administered dose	2.9 ± 2.1	508.8 ± 198.2	3.4 ± 1.9	684.1 ± 244.5
Third optimal dose	2.3 ± 1.6	412.6 ± 187.5	3.0 ± 2.1	625.0 ± 270.4
Appropriateness of administered dose compared to optimal dose	25.2%	31.6%	29.7%	28.0%

Data presented as mean ± SD of drugs' doses or % of the patients who received the appropriate dose.

*∗* Doses are based on mg/day for Valganciclovir and mg/kg/day for Ganciclovir.

*∗∗* The administered dose is considered appropriate if it was within the range of 80-120% of the optimal dose.

£ In patients receiving antiviral therapy as a part of transplantation regimen, after transplantation surgery.

**Table 3:** Patients' laboratory findings.

**Laboratory parameter (unit) **	**Value **	
	**On-admission**	**On-discharge**
Serum creatinine (mg/dL)	7.1 ± 3.1	2.1 ± 1.2
Blood urea nitrogen (mg/dL)	49.9 ± 21.0	40.5 ± 20.0
Hemoglobin (g/dL)	11.0 ± 2.2	9.3 ± 1.8
White blood cell (/mm^3^)	6.9 ± 3.2 (× 10^3^)	7.5 ± 5.3 (× 10^3^)
Neutrophil (%)	67.8 ± 11.6	83.3 ± 11.3
Red blood cell (/mm^3^)	3.8 ± 0.8 (× 10^6^)	3.3 ± 0.6 (× 10^6^)
Platelet (/mm^3^)	181.0 ± 63.7 (× 10^3^)	159.0 ± 69.5 (× 10^3^)
